# Natural polymer-based scaffolds for soft tissue repair

**DOI:** 10.3389/fbioe.2022.954699

**Published:** 2022-07-19

**Authors:** Meiwen Chen, Rui Jiang, Niping Deng, Xiumin Zhao, Xiangjuan Li, Chengchen Guo

**Affiliations:** ^1^ Hangzhou Women’s Hospital, Hangzhou, Zhejiang; ^2^ School of Engineering, Westlake University, Hangzhou, Zhejiang

**Keywords:** soft tissue repair, natural polymers, scaffolds, clinical translation, materials processing

## Abstract

Soft tissues such as skin, muscle, and tendon are easily damaged due to injury from physical activity and pathological lesions. For soft tissue repair and regeneration, biomaterials are often used to build scaffolds with appropriate structures and tailored functionalities that can support cell growth and new tissue formation. Among all types of scaffolds, natural polymer-based scaffolds attract much attention due to their excellent biocompatibility and tunable mechanical properties. In this comprehensive mini-review, we summarize recent progress on natural polymer-based scaffolds for soft tissue repair, focusing on clinical translations and materials design. Furthermore, the limitations and challenges, such as unsatisfied mechanical properties and unfavorable biological responses, are discussed to advance the development of novel scaffolds for soft tissue repair and regeneration toward clinical translation.

## Introduction

Soft tissue injury is generally caused by traumatic or pathological lesions where muscle or connective tissues get damaged (Henriksen et al., 2017). Biomaterials are used to replace the impaired soft tissue or function as scaffolds to facilitate tissue regeneration to repair the damaged soft tissue (Keane and Badylak, 2014; Gaharwar et al., 2020). Though the direct replacement of the damaged soft tissue using inert implants or autologous grafts is still commonly applied in current clinical practices, some adverse effects exist, such as chronic pain and implant-related complications (Entekhabi et al., 2021; Liang et al., 2021; Rodrigues and Raz, 2022). In comparison, scaffolds in two-dimensional or three-dimensional forms can be used as templates for tissue regeneration. The cells can bind to the scaffolds and then proliferate and differentiate (Avolio et al., 2017; Turnbull et al., 2020; Abdollahiyan et al., 2021; Bianchi et al., 2021; Masson-Meyers and Tayebi, 2021). In addition, growth factors can be incorporated into the scaffolds to advance tissue regrowth and repair (Hormozi et al., 2017; Kakudo et al., 2020; González-Pérez et al., 2021). To meet the clinical needs, the scaffolds for soft tissue repair should have tissue-matching mechanical properties, excellent biocompatibility, and appropriate biodegradability. Both synthetic polymers and natural polymers have been used to fabricate scaffolds. The synthetic polymers include polylactic acid (PLA), polyglycolic acid (PGA), poly (lactic-co-glycolic acid) (PLGA), and poly-e-caprolactone (PCL), while the natural polymers include proteins and polysaccharides (Janoušková, 2018; Rao et al., 2018). Compared with synthetic polymers, natural polymers such as collagen, fibrin, silk protein, chitosan, and hyaluronic acid generally present better biocompatibility but limited processability (Naghieh et al., 2017; Caillol, 2020; Taghipour et al., 2020). With the rapid development of processing technology in recent years, more natural polymer-based scaffolds have been successfully fabricated and applied in biomedical applications. This mini-review summarizes the status of the natural polymer-based scaffold in clinical translation and the advanced processing techniques used for making scaffolds for soft tissue repair.

## Current status of natural polymer-based scaffolds in clinical translations

Over the past decades, many natural polymer-based scaffolds for soft tissue repair have been developed for biomedical applications and some of them are commercially available. Table 1 summarizes the natural polymer-based scaffolds either commercially available or in clinical trials. These developed scaffolds are primarily composed of fibrinogen, collagen, silk, and alginate. Through advanced processing, these materials can be fabricated into functional scaffolds for various applications, including wound repair, hernia repair, cartilage repair, and blood vessel grafting. In some cases, the repairing efficacy can be improved by incorporating bioactive materials such as growth factors and antibacterial agents in the scaffolds. In recent years, silk-based scaffolds have attracted much attention due to their excellent mechanical properties and biocompatibility (Zhao and Li, 2011; Zhou et al., 2018; Mao et al., 2021; Zhao et al., 2021).

**TABLE 1:** Status of natural polymer-based scaffolds in clinical use/translation.

Trade name/Product name	Materials	Company/Institution	Applications	References
Chongshu ® composite hernia patch	Fibrinogen; poly (lactide-co-epsilon-caprolactone)	Shanghai Pine and Power Technology Co., LTD	Hernia repair	Gang et al., (2021)
Haiao ® oral repair membrane	Collagen	Yantai Zhenghai Biotechnology Co. LTD	Periodontal tissue repair	-
GenossDES™	Cobalt-chromium platform scaffolds containing sirolimus biodegradable polymers	Genoss Company Limited, Suwon, Korea	Coronary stent implantation	Lee and Park., (2020)
BEGO® collagen membrane	Collagen membrane	BEGO Implant Systems	Tissue engineering	Al-Maawi et al., (2019)
Mucograft	Collagen types I and III	Geistlich Pharma AG, Wolhusen, Switzerland	Gingival recession	Rokn et al., (2020)
Collagen Graft and Collagen Membrane	Collagen Membrane, Collagen Graf	Genoss Company Limited, Suwon, Korea	Cleft palate repair	Ha et al., (2020)
PACG-GelMA Hydrogels	Poly (N-acryloyl 2-glycine)/methacrylated gelatin hydrogels	Tianjin Key Laboratory of Composite and Functional Materials	Osteochondral Regeneration	Gao et al., (2019)
PEG silk composite hydrogel	Silk	Research Institute of Agriculture and Life Sciences, Seoul National University, Seoul, South Korea	Articular cartilage repair	Kim et al., (2021)
Elastin-silk fibroin double raschel knitted vascular graft	Silk	Tokyo University of Agriculture and Technology, Fuchu, Japan	Artificial blood vessel	Tanaka et al., (2020)
Chondrotissue®	PGA, HA	Chondrotissue, BioTissue AG, Zurich, Switzerland)	Cartilage tissue engineering	Kanatlı et al., (2017)
IC scaffold	PLGA, COL	Tissue Engineering Research Center, AIST Kansai, Amagasaki Site	Cartilage tissue engineering	Eviana Putri et al., (2020)
C2C1H scaffold	PLA, COL, CH	BioMediTech, Institute of Biosciences and Medical Technology, Tampere, Finland	Cartilage tissue engineering	Haaparanta et al., (2014)
Chitosan-modified PLCL scaffold	PLCL, CH	Tissue Engineering Program, Life Sciences Institute, National University of Singapore, Singapore	Cartilage tissue formation	Yang et al., (2012)
CSMA/PECA/GO (S2) scaffold	CSMA, MPEG-PCL-AC (PECA), GO	State Key Laboratory of Biotherapy and Cancer Center, West China Hospital, Sichuan University	Cartilage tissue engineering	Liao et al., (2015)
Hyalofast®	Benzyl ester of hyaluronic acid	Anika Therapeutics Inc., Bedford, Massachusetts, United States	Osteochondral Injury	Bajuri et al., (2021)
ChondroGide®	Type I/III collagen	Geistlich Biomaterials, Wolhusen, Switzerland	Cartilage defects of the knee joint	Niemeyer et al., (2008)
Cartipatch®	Agarose and alginate	Tissue Bank of France, TBF, Lyon, France	Knee cartilage injury	Clavé et al., (2016)
Silk Voice®	Silk	Sofregen, United States	Wound healing	-
NOVOCART® 3D	Type I collagen, chondroitin sulfate	TETEC, Reutlingen, Germany	Isolated retro patellar cartilage defects	Kayaalp et al., (2021)

CH, chitosan; COL, collagen; CSMA, methacrylated chondroitin sulfate; HA, hyaluronic acid; PCL, polycaprolactone; PLA, polylactic acid; PLLA, poly (l-lactide); PGA, poly (glycolic acid); PLGA, polylactic-co-glycolic acid; ECM, extracellular matrix; PLCL, poly (l-lactide-co-ε-caprolactone); AC, acryloyl chloride; GO, graphene oxide.

## Fabrication of natural polymer-based scaffolds

An ideal scaffold for soft tissue repair should meet the requirements for specific applications, including good biocompatibility, suitable mechanical properties, satisfied porosity, and controlled degradability. (Arbade et al., 2020) (Janoušková, 2018) The satisfied pore size for soft engineering is ∼5–200 μM. (Janoušková, 2018) Regarding the mechanical properties, constructing scaffolds with matching mechanical properties to native soft tissues is very critical. Human tissues span a broad spectrum of mechanical properties, where stiffness of soft tissues typically ranges from 1 kPa (e.g., brain) to ∼1 MPa (e.g., nerve and cartilage). (Guimarães et al., 2020) Over the past decades, numerous approaches have been developed for fabricating natural polymer-based scaffolds, such as electrospinning, freeze-drying, and 3D printing. In this section, we provide a general overview of these approaches and discuss their use in processing natural polymers into functional scaffolds.

### Electrospinning

**FIGURE 1:**
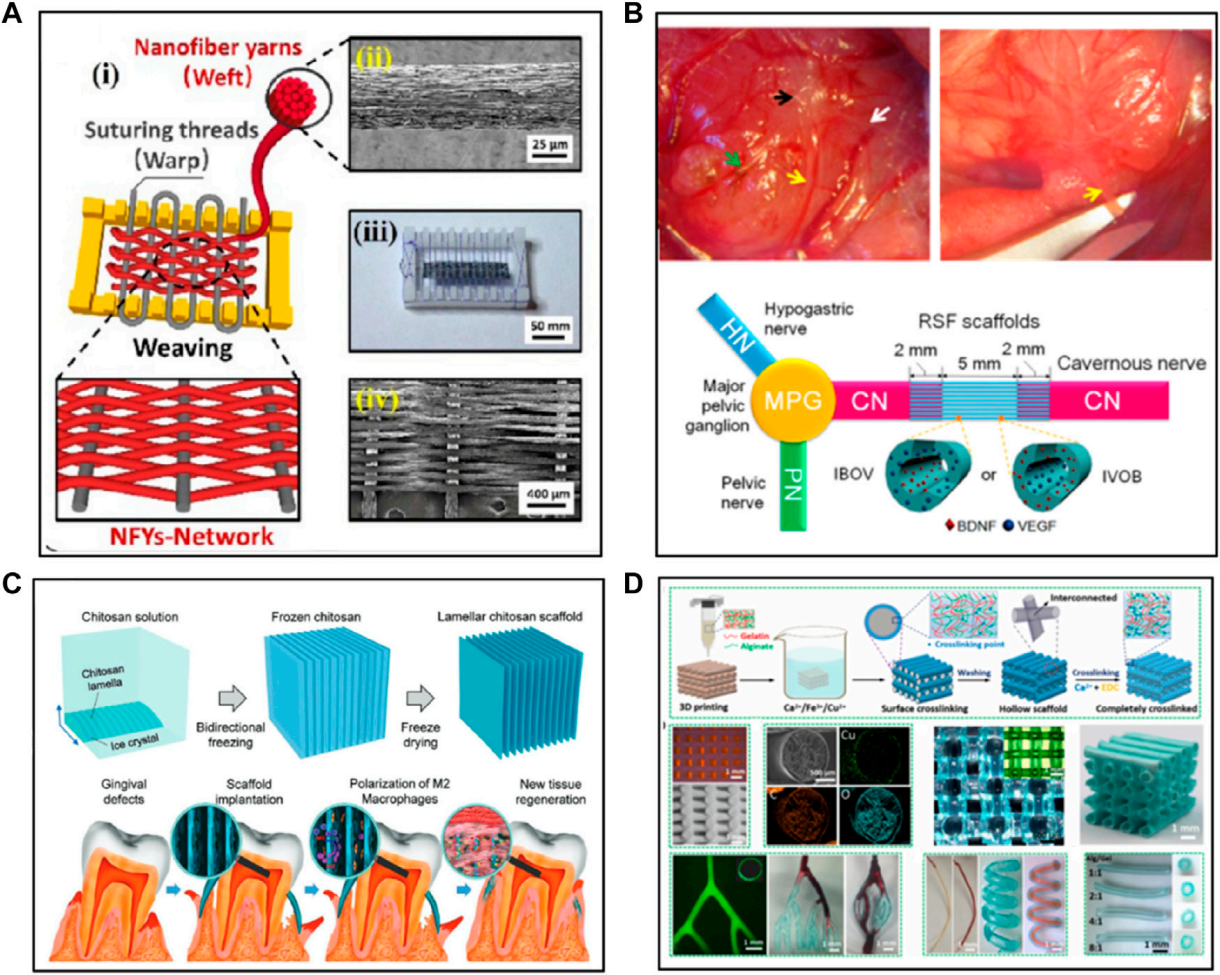
The fabrication of natural polymer-based scaffolds via various methods: **(A,B)** Electrospinning. **(C)** Freeze-drying and **(D)** 3D printing. The pictures got permissions from (Zhang et al., 2016), (Wu Y. et al., 2017), (Feng et al., 2021), (Luo et al., 2022), respectively.

Electrospinning offers a convenient approach to fabricate fiber-based scaffolds for soft tissue repair. Nanofibers can be fabricated through electrospinning from polymer solutions under a high electrical field and further organized into porous nanofiber-based mats. When designing an ideal electrospun scaffold for soft tissue repair, some critical factors need to be considered. These factors include biocompatibility, mechanical properties, porosity, and the ability to regulate cellular behavior (Zhong et al., 2022). Many studies have been reported on fabrication of natural polymer-based scaffolds using electrospinning. Lee et al. fabricated electrospun nanofibrous gelatin sheets and investigated the influence of electron beam (e-beam) irradiation doses on the molecular weight, morphology, pore structure, and cell proliferation profiles of the sheets (Lee et al., 2017). In addition, electrospinning using a core-shell nozzle was employed to make collagen/polyvinylpyrrolidone (PVP) core-shell nanofibers where the collagen was encapsulated within a shell of PVP. The PVP shell was then washed away in a basic ethanol solution to yield anisotropic collagen nanofibers which mimics the structures of the native extracellular matrix (Wakuda et al., 2018). It is worth mentioning here that the structure of the nanofiber-based mats mimics the structure of the natural extracellular matrix, providing a biomimetic microenvironment for cells to proliferate and differentiate (Nie et al., 2020). Moreover, some strategies have been developed to enhance the physical properties and biofunctions of the scaffolds. These scaffolds have been widely used in several soft tissue engineering, such as skin, vascular tissue, cavernous nerve (CN) and cardiac tissues (Ehrmann, 2021). For instance, Uibo et al. demonstrated that the scaffold composed of salmon fibrinogen and chitosan could promote wound healing without any complications (Laidmäe et al., 2018). Jadbabaei et al. developed a novel approach to enhance the electrospinnability of sodium alginate and made alginate-PVA polymeric scaffolds for skin tissue engineering applications (Jadbabaei et al., 2021). Zhang et al. successfully fabricated silk-based scaffolds for cavernous nerve (CN) regeneration using coaxial electrospinning. The scaffolds with a core of RSF-VEGF and a shell of RSF-BDNF promoted the regeneration of cavernous nerves and were able to converse into nerve guidance conduit to facilitate nerve regeneration (Figure 1A) (Zhang et al., 2016). Recombinant spider silk protein (pNSR32) and gelatin (Gt) were also used to enhance the cytocompatibility of electrospun PCL scaffolds. The pNSR32/PCL/Gt composite scaffolds show potential for small-caliber vascular tissue engineering (Xiang et al., 2018). Wu et al. designed a scaffold for cardiac tissue regeneration to guide the orientation of the cells by mimicking the anisotropic cardiac structure (Wu Y. et al., 2017). The scaffold within a hydrogel shell was composed of aligned electrospun conductive nanofibers (NEYs-NET) which contained the polycaprolactone, silk fibroin, and carbon nanotubes. Cardiomyocytes (CMs) were aligned along the nanofibers on each layer of the 3D nanofibrous scaffold in the stable hydrogel environment (Figure 1B). Overall, the electrospinning technique allows researchers to fabricate ECM-mimic nanofibrous scaffolds with tunable fiber diameters, surface areas, porosity depending on different technique factors, such as solution viscosity and work voltage. Furthermore, electrospinning provides the feasibility for introducing and incorporating bioactive molecules for soft tissue repair and regeneration (Arbade et al., 2019).

### Freeze-drying

Freeze-drying is an easy and eco-friendly method that can be readily used to fabricate 3D scaffolds with microporous structures. For fabrication of natural polymer-based scaffolds, natural polymers were first dissolved in water to obtain aqueous solutions, followed by freeze-drying. During freeze-drying, the water in the frozen sample undergoes sublimation under a high vacuum, leading to the scaffolds with porous structures. The pore size in the scaffolds depends on the type of natural polymers and the concentration of the solution. The porous structures generated via freeze-drying benefit the cells to attach, differentiate, proliferate, and mass transport. Indurkar et al. showed that the physical parameters of the scaffolds, such as surface roughness, porosity, interconnectivity, and contact angle influence the transport of nutrition and waste products (Indurkar et al., 2020). Furthermore, Afjoul et al. prepared alginate-gelatin scaffolds through freezing dry and revealed that the ratio of alginate to gelatin affects swelling, biodegradation, cell culture, and mechanical properties of the scaffolds. The optimized scaffolds showed good biocompatibility and satisfied outcomes of wound healing in rats (Afjoul et al., 2020). In another study, Chen et al. prepared a hybrid cobalt-doped alginate/waterborne polyurethane 3D porous scaffold with nano-topology of a “coral reef-like” rough surface via two-step freeze-drying (Chen et al., 2021). The “coral reef-like” rugged surface topology and bioactive cobalt dopant synergistically promote the neurite outgrowth and up-regulate the synaptophysin expression of neuron-like cells PC12 on the scaffold. In addition, two types of cellulose-derived materials, oxidized cellulose and carboxymethyl cellulose (CMC), were mixed with collagen to fabricate scaffolds through freeze drying. The prepared scaffolds showed good mechanical properties, hemostasis, and antibacterial properties (Kacvinská et al., 2022). Protein-based scaffolds have also been developed. For example, dual-crosslinked silk fibroin scaffolds with EGDE have been developed, where the researchers showed that an appropriate dosage of crosslinking agent was critical to achieve good mechanical properties, *in vivo* degradability, and mild immune responses in soft tissue engineering (Mao et al., 2021). The scaffold notably relieved the inflammatory response of microglial cells BV2 with the transformation from pro-inflammatory (M1) to anti-inflammatory (M2) phenotype. Regarding better control of the structure, morphology, and density of scaffolds, Jiang et al. developed chitosan scaffolds with tunable microchannels by combining a 3D printing-assisted microfiber templates-leaching approach and a freeze-drying approach (Jiang et al., 2021). Moreover, Feng et al. fabricated a novel chitosan scaffold with lamellar structures by mimicking the layered structure of the attached gingiva using a bidirectional freeze-drying method (Feng et al., 2021). The bio-inspired lamellar chitosan scaffold (LCS) with ordered porous structure showed excellent mechanical properties, good cell-compatibility and could promote the vessel formation and gingival tissue regeneration *in vivo*. In addition, the LCS is found to be capable of inducing macrophage differentiation to M2 macrophages, which is thought to play an important role in tissue regeneration (Figure 1C). Also, the microstructure of the scaffolds could be controlled by optimizing the mold and freezing parameters for a certain application. Brougham et al. developed organ-specific collagen-based scaffolds geometries for tissue engineering applications, where the geometries of the scaffolds could be tailored by adjusting the mold patterns and freezing parameters (Brougham et al., 2017). In a brief summary, freeze-drying is a good method for natural polymer-based scaffold fabrication since it is easily applied to obtain porous structures without a high temperature or a washing step though the fabrication time is relatively long (Boffito et al., 2014).

### 3D printing

Three-dimensional (3D) printing is a technique that can be used to fabricate biomedical scaffolds in a controlled way (Pérez-Köhler et al., 2021). Compared with traditional thermal-based 3D printing, 3D bioprinting combines 3D printing with living cells or other non-living biological materials (e.g., growth factors, drugs) to construct scaffolds for tissue engineering and tissue regeneration. 3D bioprinting allows researchers to design 3D tissue-mimicking scaffolds which provide tailored cellular environments to facilitate the growth and proliferation of cells (Kim et al., 2016; Perez-Puyana et al., 2020). A broad range of natural polymer-based scaffolds have been fabricated using 3D bioprinting and some studies have been reported. Regarding the materials used for making the bioinks, a variety of materials have been used including collagen, gelatin, alginate, silk fibroin, and extra cellular matrix (ECM). For example, Jang et al. fabricated the artificial skin based on decellularized ECM derived from porcine skin via 3D bioprinting method (Jang et al., 2021). The 3D printed artificial skin exhibited rapid re-epithelialization and facilitate tissue regeneration on a mouse chimney wound model, showing great potential of clinical translation. In addition, alginate-based scaffolds with the features of high cell viability and low concentration alginate for potential nerve tissue engineering application were developed (Naghieh et al., 2019). Moreover, Tijore et al. developed a 3D bioprinting microchannel gelatin hydrogel that promoted human mesenchymal stem cells (hMSCs) myocardial commitment and supports native cardiomyocytes (CMs) contractile functionality (Tijore et al., 2018). Luo et al. used gelatin and alginate to fabricate scaffolds with microporous structures and interconnected microchannels using 3D bioprinting (Figure 1D) (Luo et al., 2022). The fabricated scaffold could support vascularization and growth of new tissues, promoting wound healing. Furthermore, Wang et al. fabricated a hybrid hydrogel system using a combination of decellularized extracellular matrix (dECM-G) and photo-crosslinkable gelatin methacrylate (GelMA) for nerve regeneration (Wang et al., 2022). The system showed good printability and structural fidelity for facilitating neurite growth and cell migration. Fabrication of scaffolds with designed microstructures to guide cell growth also attracts a lot of attention recently. Wu et al. precisely controlled architectures of micro-structured and stretchable chitosan hydrogels for guided cell growth (Wu Q. et al., 2017). The hybrid bioink prepared with gelatin, sodium alginate, and carbon nanotubes were used to fabricate cylindrical scaffolds through a combination of the vertical directional extrusion of printing nozzle and axial rotation of stepper motor module for blood vessel regeneration (Li et al., 2020).

## Challenges and opportunities

Natural polymer-based scaffolds have been rapidly developed and applied in soft tissue repair in the past few decades. Some products are now commercially available and in clinical use. However, some limitations are associated with the current products, such as unsatisfied mechanical properties, uncontrolled degradability, and unfavorable immune response. Some critical points need to be considered when developing high-performance scaffolds that better meet clinical needs. Firstly, advanced processing approaches are required to achieve high-quality processing of natural polymers. For collagen-based materials, how to maintain their bioactivity during processing is a challenge. Secondly, rational materials design and advanced fabrication technologies are needed since the structures and properties of the scaffolds should be tailored for different applications. For instance, for treating pelvic organ prolapse, porous scaffolds with robust mechanical properties and controlled biodegradability are required. In some applications, an aligned scaffold is preferred to allow the cells to grow directionally. Moreover, enhancing the biocompatibility and mimicking the biological functions of the extracellular matrix should be considered. Integrins and cadherins can be grafted to the scaffolds since they are serving as adhesion molecules for migration and localization of cells. Furthermore, patient-oriented scaffold design with the assistance of the 3D printing fabrication technique is of great potential to offer precise repair. Thirdly, scaffolds with bioresponsiveness or biofunctions are promising since such scaffolds allow better tissue repair control. For example, scaffolds with the incorporation of antibiotics can effectively prevent infections during the tissue regeneration process. In addition, growth factors can be incorporated into scaffolds to facilitate tissue repair. Lastly, a comprehensive understanding of the materials-cell interactions is needed to support the development of novel functional scaffolds. The fundamental research would lay a solid foundation for novel material designs, the development of advanced fabrication techniques, and clinical translations.
