# Supplementary material for: Natural polymer-based scaffolds for soft tissue repair
Source: Front Bioeng Biotechnol. 2022 Jul 19;10:954699. doi: 10.3389/fbioe.2022.954699 (PMC9343850; doi:10.3389/fbioe.2022.954699)
Supplement: Supplementary file 1 [file DataSheet2.PDF]

Silk Fibroin-Based Scaffolds with Controlled Delivery Order of VEGF and BDNF for Cavernous Nerve Regeneration

Author: Yaopeng Zhang, Jianwen Huang, Li Huang, et al
Publication: ACS Biomaterials Science & Engineering
Publisher: American Chemical Society
Date: Nov 1, 2016

Copyright © 2016, American Chemical Society

PERMISSION/LICENSE IS GRANTED FOR YOUR ORDER AT NO CHARGE

This type of permission/license, instead of the standard Terms and Conditions, is sent to you because no fee is being charged for your order. Please note the following:

- Permission is granted for your request in both print and electronic formats, and translations.
- If figures and/or tables were requested, they may be adapted or used in part.
- Please print this page for your records and send a copy of it to your publisher/graduate school.
- Appropriate credit for the requested material should be given as follows: "Reprinted (adapted) with permission from {COMPLETE REFERENCE CITATION}. Copyright {YEAR} American Chemical Society." Insert appropriate information in place of the capitalized words.
- One-time permission is granted only for the use specified in your RightsLink request. No additional uses are granted (such as derivative works or other editions). For any uses, please submit a new request.

If credit is given to another source for the material you requested from RightsLink, permission must be obtained from that source.

BACK CLOSE WINDOW

© 2022 Copyright - All Rights Reserved | Copyright Clearance Center, Inc. | Privacy statement | Terms and Conditions
Comments? We would like to hear from you. E-mail us at customercare@copyright.com
